# Exercise and bone health across the lifespan

**DOI:** 10.1007/s10522-017-9732-6

**Published:** 2017-10-20

**Authors:** Lívia Santos, Kirsty Jayne Elliott-Sale, Craig Sale

**Affiliations:** Musculoskeletal Physiology Research Group, Sport, Health and Performance Enhancement Research Centre, School of Science and Technology, Nottingham Trent University, Nottingham, NG11 8NS UK

**Keywords:** Exercise, Lifespan, Bone health, Bone adaptation, Bone ageing, Osteoporosis

## Abstract

With ageing, bone tissue undergoes significant compositional, architectural and metabolic alterations potentially leading to osteoporosis. Osteoporosis is the most prevalent bone disorder, which is characterised by progressive bone weakening and an increased risk of fragility fractures. Although this metabolic disease is conventionally associated with ageing and menopause, the predisposing factors are thought to be established during childhood and adolescence. In light of this, exercise interventions implemented during maturation are likely to be highly beneficial as part of a long-term strategy to maximise peak bone mass and hence delay the onset of age- or menopause-related osteoporosis. This notion is supported by data on exercise interventions implemented during childhood and adolescence, which confirmed that weight-bearing activity, particularly if undertaken during peripubertal development, is capable of generating a significant osteogenic response leading to bone anabolism. Recent work on human ageing and epigenetics suggests that undertaking exercise after the fourth decade of life is still important, given the anti-ageing effect and health benefits provided, potentially occurring via a delay in telomere shortening and modification of DNA methylation patterns associated with ageing. Exercise is among the primary modifiable factors capable of influencing bone health by preserving bone mass and strength, preventing the death of bone cells and anti-ageing action provided.

## Introduction

Ageing is accompanied by the loss of bone mass and strength, predisposing the skeleton to the onset of osteoporosis (Demontiero et al. 2012). Osteoporosis is a metabolic disorder prevalent in post-menopausal women, characterised by accentuated bone weakening, greater susceptibility to fragility fractures (Hernlund et al. 2013), but also higher mortality risks (Klop et al. 2017; Panula et al. 2011). Hip fractures and associated comorbidities in particular, are responsible for the increase in 1-year mortality risks by more than threefold when compared with those without a bone fracture (Klop et al. 2017; Panula et al. 2011).

Osteoporosis is estimated to affect 22 million women and 5.5 million men in the EU (Hernlund et al. 2013). In 2010, there were 3.5 M osteoporotoic fractures reported in the EU; 620,000 hip fractures, 520,000 vertebral fractures, 560,000 forearm fractures and 1,800,000 other fractures (Hernlund et al. 2013). In the UK, 3.21 M people, aged over 50 years, are living with osteoporosis, with more than 536,000 new fragility fractures occur every year (Svedbom et al. 2013). The prevalence of osteoporosis is expected to rise over the next decades by virtue of population ageing. One-third of the UK population is 50 years old or above, and current estimates suggest this age segment will grow from 21.6 million in 2010 to 26.2 million in 2025, corresponding to an increase of 21% (UK Office for National Statistics 2016).

Osteoporosis and other musculoskletal disorders, particularly osteoarthritis and bone trauma, are amongst the most common problems affecting the elderly and are a leading cause of physical disability (Gheno et al. 2012; Weinstein 2016). Limitations in mobility and independance are psychologically devastating and represent a huge economic challenge to the sustainability of health care systems (Gheno et al. 2012; Weinstein 2016). Exercise, nutrition and pharmacological interventions may help the management of age-related bone loss and osteoporosis. Certain types of exercise might result in improved bone strength even after menopause, a time when bone mass declines and the ability to rescue lost bone is impaired (Polidoulis et al. 2011; Uusi-Rasi et al. 2003). With regard to nutrition, vitamin D is essential in calcium metabolism and oral intake may prevent fractures in osteoporotic patients (Lips et al. 2006). Pharmacological interventions are the gold standard with regards to osteoporosis management and prevention of fragility fractures, although their benefits are transient and might induce rare but severe side effects (Gozansky et al. 2005; Woo et al. 2006). Some of the concerns raised as a result of these side effects might well have contributed to the declining prescription of these drugs or the reduction in actual use of prescribed medications for low bone mass (Jha et al. 2015).

Exercise is one of the primary modifiable factors associated with improved bone health outcomes, such as high bone mineral density (BMD) and strength (Weaver et al. 2016). Individuals who undertake exercise on a regular basis are also more likely to prevent age-relate bone loss and experience fewer falls and fractures by virtue of developing stronger muscles and bones, which improve balance (Liu-Ambrose et al. 2004). In addition to this, exercise may provide a “rejuvenating effect” and, as a result, the potential to mitigate age-related bone loss and diseases (Loprinzi et al. 2015). In this article, we review the benefits of undertaking exercise throughout life as part of a strategy to promote bone health across the lifespan, and advance some cellular and molecular mechanisms potentially activated upon exercise that underpin such benefits. We will also highlight some areas where the clinical benefits of exercise on bone health might have been slightly exaggerated, given that increases in bone mass as a result of exercise are typically in the range of 1–10% at the most and reductions in bone mass across the lifespan are significantly greater (Riggs et al. 2004).

Bone and muscle are the two largest tissues of the musculoskeletal system and they are coupled mechanically, biochemically and molecularly (Brotto and Bonewald 2015), with muscular contraction thought to be the main source of mechanical strain leading to bone adaptation (Bakker et al. 2001; Burr 1997). Bone and muscle mass/strength are proportionally related, as evidenced by a study showing that under disuse conditions, muscle mass declines followed by a loss of bone mass, while during recovery muscle mass gains precede bone accretion (Sievänen et al. 1996). Although coupling between the two tissues and further interactions with other elements of the musculoskeletal system, particularly tendons, ligaments and cartilage is unquestionable, particularly in relation to the prevention of falls (perhaps the major contributor to bone fracture), this is beyond the scope of the present review.

## Ageing and bone loss

### Ageing

Ageing is a physiological process that results from the accumulation of molecular and cellular damage over time (WHO 2015). It is influenced by the human genome and epigenetic changes triggered by environmental and lifestyle factors (Govindaraju et al. 2015). Human ageing is generally accompanied by a decline of cognitive and motor functions (Moustafa 2014) and is considered the main risk factor for developing musculoskeletal, neurodegenerative and cardiovascular diseases (Niccoli and Partridge 2012). Genetic studies on progeroid syndromes, clinical conditions of premature ageing, have been useful to understand physiological ageing and age-related diseases (Martin and Junko 2010). Research on Hutchison-Gilford and Werner progeroid syndromes, in particular, have allowed the identification of several hallmarks of physiological ageing, such as telomere shortening, mitochondrial dysfunction, oxidative stress and cell senescence (Childs et al. 2015; López-Otín et al. 2013). Briefly, telomeres are protective caps located at the end of chromosomes with the purpose of preventing deterioration or fusion with other chromosomes. Telomere shortening exacerbates human ageing, as well as inducing metabolic alterations, such as insulin resistance, β-cell failure and glucose intolerance (Gardner et al. 2005; Shimizu et al. 2014). The mitochondria are organelles that generate the majority of the chemical energy utilised by cells, adenosine triphosphate (ATP). Mitochondrial dysfunction is caused by depletion of nicotinamide adenine dinucleotide (NAD+) and downregulation of the tricarboxylic acid and oxidative phosphorylation (OXPHOS) pathways (Zhang et al. 2016), leading to a decline in respiratory function and stem cell senescence (Wiley et al. 2016; Zhang et al. 2016). Senescent cells exhibit stress-induced permanent proliferative arrest and are thought to drive ageing and age-related pathologies (Baker et al. 2016; Childs et al. 2015). While in proliferative arrest, senescent cells secrete specific proteins, referred to as the senescence-associated secretory phenotype (SASP), which can exacerbate the proliferative arrest and also induce senescence in a paracrine manner. Interestingly, recent evidence came to light showing that the SASP can also exert a proregenerative effect through cell plasticity and stemness (Ritschka et al. 2017). Lastly, excessive or persistent oxidative stress caused by the action of free radicals, non-ionising radiation and inflammatory agents, and from mitochondrial by-products (e.g., peroxides), was proposed to contribute to accumulated DNA damage and activation of apoptotic signalling pathways, potentially accelerating ageing (Kryston et al. 2011; Lu et al. 2012). Oxidative stress was identified as an important driver of bone ageing. This marker will be further discussed in the next section (Ambrogini et al. 2010; Manolagas 2010).

### Age-related bone loss

**Fig. 1:**
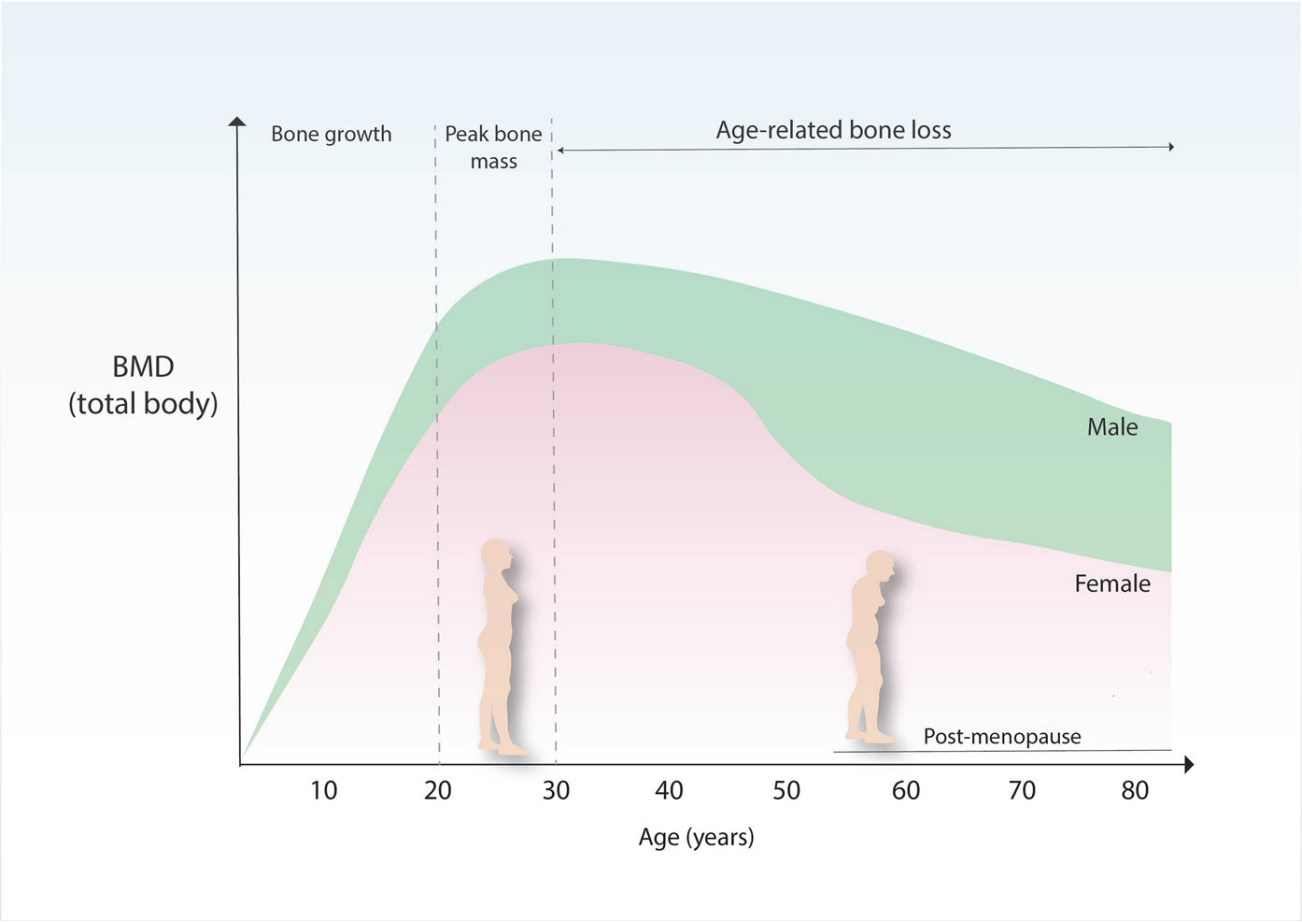
Bone mass density (BMD) across the lifespan. Men exhibit higher BMDs throughout life and are less susceptible to age-related bone loss than women. Adapted from Hendrickx et al. (2015)

Bone accretion occurs from birth and throughout childhood and adolescence, with approximately 90% of bone mass acquired by the age of 20 years (Henry et al. 2004; Recker et al. 1992). Acquisition of bone mass follows sex and age specific patterns, as evidenced in Fig. 1. Men have greater BMD than women and this difference becomes starker as sexual maturation progresses (Hendrickx et al. 2015). When women reach late 30s and men early 40s, BMD starts to decline and this trend persists throughout life (Fig. 1). Such decline is further accompanied by a decrease in bone strength strength (Wall et al. 1979), osteocyte death, deteoration of type I collagen (Bailey and Knott 1999) and adipogenesis at the expense of osteogenesis (Justesen et al. 2001). Age-related bone loss occurs due to greater bone resorption than bone formation, a process that culminates in reduced trabecular volume and diminished cortical bone width (McCalden et al. 1993). For a comprehensive review of these changes see (Boskey and Coleman 2010; Manolagas and Parfitt 2010).

Oxidative stress has been identified as a critical driver of bone ageing (Ambrogini et al. 2010; Manolagas 2010). Production of mitochondrial superoxide anion (O_2_^−^) in aged osteocytes led to increased osteoclast-mediated bone resorption (Kobayashi et al. 2015). In addition to this, the presence of reactive oxygen species (ROS) has been shown to attenuate β-catenin signalling with concomitant activation of PPARγ favouring adipogenesis at the expense of osteoblastogenesis and bone formation (Manolagas 2010). The loss of function of oxidative defense Forkhead box O (FOXOs), a family of genes implicated in ageing and longevity, triggers the apoptosis of osteoblasts and osteocytes and the advance of an osteoporotic phenotype (Ambrogini et al. 2010). In this same study, the authors showed that an overexpression of FOXO3 in osteoblasts culminated in increased bone mass. These findings demonstrate that signalling pathways implicated in bone cell survival and osteogenesis are negatively affected by oxidative stress leading to age-related bone loss and potentially osteoporosis.

## The osteoporotic bone

Osteoporosis is the most prevalent disease in post-menopausal women and is accompanied by an increased risk of fragility fractures (Ji and Yu 2015). Fragility fractures occur primarily in the spine, hip and wrist (NICE 2012). Hip fractures cause permanent disability in 50% of the cases and death in 20% (Sernbo and Johnell 1993). In the UK, 300,000 fragility fractures occur every year (British Orthopaedic Association 2007), with direct medical costs estimated at £1.8 billion in 2000 and projected to reach £2.2 billion by 2025 (Burge et al. 2001).

Osteoporosis arises from the imbalance between bone resorption (osteoclast-mediated) and bone formation (osteoblast-mediated), with bone resorption exceeding bone formation. At histopathological level, the osteoporotic bone is less compact as a result of bone thining or loss, presents a strong reduction in the trabecular connectivity and greater adiposity of the bone marrow (Marcu et al. 2011).

Oxidative stress and oestrogen depletion are two important mechanisms underpinning osteoporosis. Oxidative stress was reported to direct commitment of mesenchymal progenitors towards the adipogenic lineage at the expense of osteoblastogenesis (Manolagas 2010), which can explain greater adipodicity of the bone marrow in old and osteroporotic bone (Justesen et al. 2001). Oestrogen has a protective role in bone health e.g., by controlling bone resorption activity. This was demonstrated by studies evidencing that oestrogen inhibits osteoclast formation and activity via increased production of osteoprotegerin (Hofbauer et al. 1999) or transforming growth factor β (Hofbauer et al. 1999; Hughes et al. 1996), and may also induce apoptosis of osteoclast progenitor cells via blocking of the cytokine receptor activator of NFκB ligand (RANKL) (Lundberg et al. 2001). Oestrogen action on bone resorption activity was further confirmed by a study showing that selective deletion of the oestrogen receptor-α (ERα) in osteoclast lineage cells increased osteoclastogenesis activity and abrogated the oestrogen-mediated pro-apoptotic action in osteoclasts (Almeida et al. 2013). These changes led to increased bone resorption in women, but not in men, causing a loss of cancellous, but not cortical, bone (Almeida et al. 2013). When oestrogen is depleted in the organism, e.g., post-menopause, this protective effect on bone health is reduced or disappears and this increases predisposition to the onset of bone diseases like osteoporosis.

Osteoporosis is conventionally appraised by dual-energy X-ray absorptiometry (DXA) and the resultant BMD values are compared to the BMD of young healthy individuals of the same gender, thus generating a *T* score. A *T* score of −1 and above is considered normal, a score between −1 and −2.5 is indicative of osteopenia, and a score of −2.5 or below signifies osteoporosis. This categorisation was established by the Word Health Organisation (WHO) to standardise the diagnosis of oesteoporosis, particularly in Caucasian, postmenopausal women. BMD values can also be compared to the BMD of age-matched individuals with normal bone mass to generate a *Z* score. *Z* Scores are mostly utilised in cases of severe osteoporosis. BMD is, however, only one element of bone strength, with areal BMD (aBMD) accounting for 65–75% of the variance in bone strength. As such, there is a need to also consider volumetric BMD, bone geometry and bone architecture.

According to the severity of bone loss, the presence of fragility fractures and other clinical factors, patients may be prescribed with anti-osteoporotic drugs, primarily the oral intake of bisphosphonates, such as alendronate. Third generation (nitrogen-containing) alendronate binds to bone mineral and is metabolised by osteoclasts leading to the inhibition of bone resorptive activities and an increase of bone strength (Boivin et al. 2000). Another important anti-bone resorption drug is strontium ranelate, although its mechanism of action differs from bisphosphonates by targeting bone formation and mineralisation directly, rather than by suppressing osteoclast-mediated bone resorption activity (Marie 2007). Denosumab is a human monoclonal antibody that binds to RANKL, inhibiting it. RANKL suppression impairs osteoclast maturation and survival leading to the diminution of bone resorption activity (Hanley et al. 2012). The teriparatide human recombinant parathyroid hormone (hrPTH), is clinically approved for the treatment of osteoporosis due to its anabolic effect on bone and its ability to rescue skeleton strength (Pazianas 2015). The use of hrPTH is recommended for up to 24 months and has been shown to reduce fracture risks (Lindsay et al. 2016; Neer et al. 2001).

The prescription of anti-osteoporotic drugs is vital for the management of osteoporosis and its related co-morbidities, although they are not always effective and the benefits are transient (Gozansky et al. 2005). Gozansky et al. (2005) investigated the efficacy of oestrogen and raloxifene in conserving BMD during a 6-month exercise-based weight loss program (Gozansky et al. 2005), where participants were allowed to select the mode(s) of exercise e.g., treadmill, walking/running, cycling, among others. The authors showed that both pharmacological interventions failed to maintain intact lumbar spine, total hip and trochanter BMD in post-menopausal women enrolled in a lost weight program, although BMD losses were more pronounced in women belonging to the placebo group (Gozansky et al. 2005). With regard to side effects, long-term use of bisphosphonates can cause severe collateral damage, such as jaw necrosis (Woo et al. 2006). In light of this, it has been advocated that regular exercise might be one of the best non-pharmacological approaches to support bone health across the lifespan (Gomez-Cabello et al. 2012), either by maximising peak bone mass during maturation, delaying the onset of osteoporosis later in life (Tveit et al. 2015; Warden et al. 2007) and/or by mitigating the age and/or menopausal-related bone loss (Howe et al. 2011; Polidoulis et al. 2011). Much of the evidence in support of a positive effect of exercise on bone is, however, observational and many of the direct exercise intervention studies have not shown such large effects on bone. Over the next sections the influence of exercise on age-related bone loss and osteoporosis will be discussed.

## Bone remodelling and adaptation to exercise

Bone is a heterogeneous tissue made up of two components, an organic part comprised of collagenous and non-collagenous proteins and cells and a mineral component of hydroxyapatite (Boskey 2013). Bone contains three major cell types: osteoblasts, which derive from mesenchymal stem cells and are responsible for bone formation; osteocytes, dendritic cells terminally differentiated from osteoblasts embedded in the bone matrix, accounting for more than 90% of bone cells; and osteoclasts, large multinucleated cells differentiated from hematopoietic progenitor cells that mediate bone resorption (Schaffler et al. 2014; Tatsumi et al. 2007). The coordinated action of osteoblasts, osteoclasts and osteocytes orchestrate bone modelling and remodelling. Bone modelling occurs to accommodate the radial and longitudinal growth of bone during the growing years and to adapt the skeleton to mechanical strain, whereas remodelling happens mainly during adulthood to remove microdamaged and old bone, adapt bone tissue to mechanical loading and maintain the strength and integrity of the skeleton (Sims and Martin 2014). During modelling, osteoclastogenesis and osteogenesis work independently, whereas in remodelling, bone resorption and formation are coupled, taking place in bone remodelling units (Baron and Kneissel 2013).

### Bone adaptation to exercise

Exercise leads to bone adaptation and this process is mediated by cellular mechanotransduction (Goodman et al. 2015). Briefly, upon exercise, bone tissue deforms, and the mechanosensors located throughout the cells, such as stretch-activated ion channels and integrins, change their original conformation (Guilluy et al. 2014; Ross et al. 2013). Such conformational changes trigger a signalling cascade to provide an appropriate biochemical response (Goodman et al. 2015), e.g., osteogenesis and bone accretion at the site of deformation.

Osteocytes are mechanotransduction hot spots due to their unique ability to detect and respond to mechanical strains (Klein-Nulend and Bakker 2007). Osteocytes control bone formation and resorption through the differentiation of osteoblasts and osteoclasts and by stimulating the expression of the osteoclastogenesis inhibitor, osteoprotegerin (Regard et al. 2012). Osteoblasts also secrete osteoprotegerin evidencing that this cell type also presents the potential to regulate bone resorption activity (Udagawa et al. 2000).

**Fig. 2:**
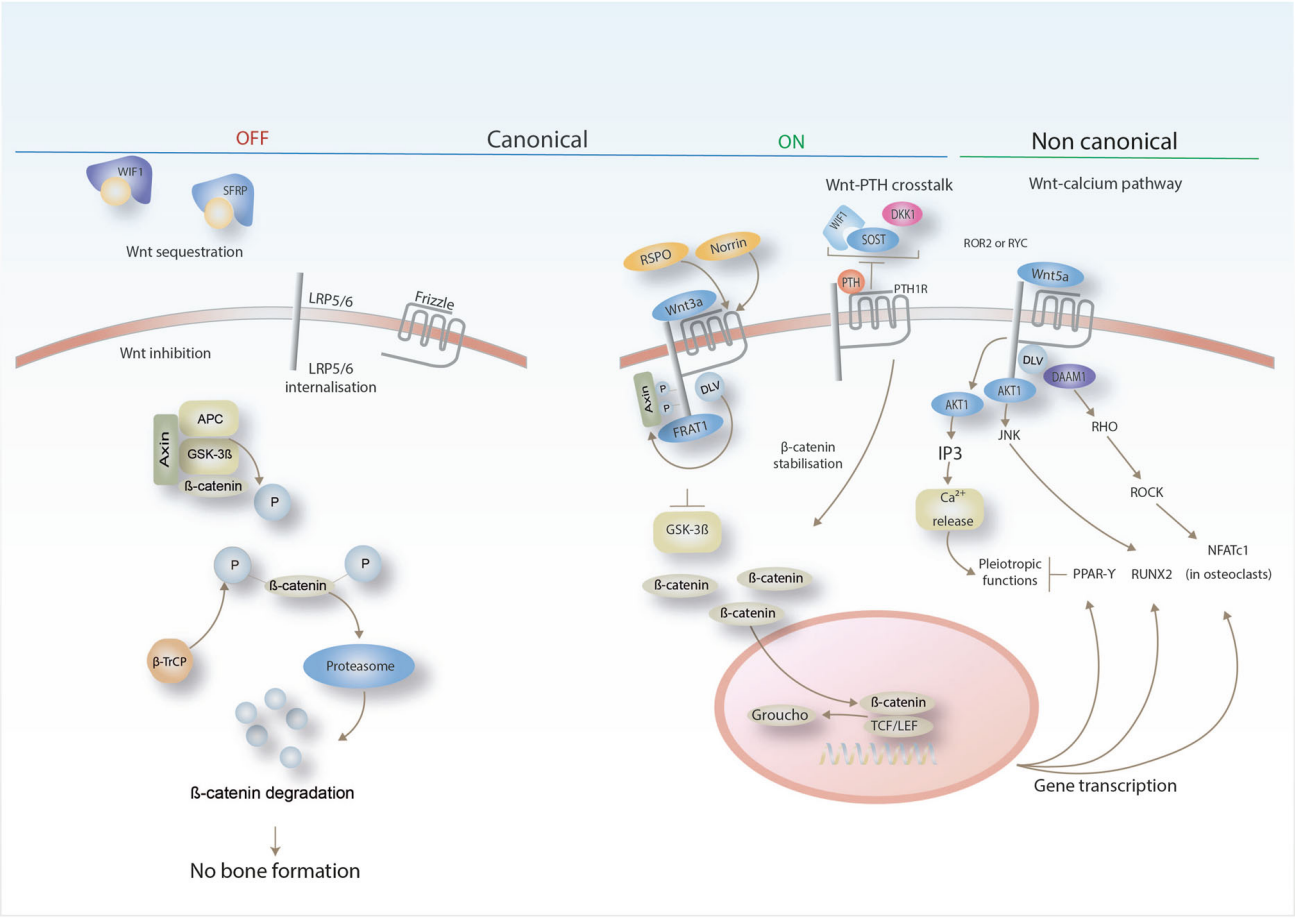
Simplified diagram depicting canonical and non canonical β-catenin signalling pathways in bone. Exercise enables bone formation through the active canonical and non-canonical β-catenin signalling pathways. Activation of the bone transcription factor RUNX2 elicits osteogenesis and supresses PPAR-γ-mediated adipogenesis; Activation of WIF1: Wnt Inhibitory Factor 1: SFRP: Secreted frizzled-related protein; LRP5/6: Low-density lipoprotein receptor-related protein 5/6; APC: adenomatous polyposis coli; GSK-3β: glycogen synthase kinase 3 beta; Ub: ubiquitination; P: phosphorylation; β-TrCP: beta-transducin repeat containing E3 ubiquitin protein ligase; RSPO: R-spondin 1; WNT3A: Wnt family member 3A; FRAT1: FRAT1, WNT signalling pathway regulator; DVL: dishevelled segment polarity protein 1; TCF/LEF: T cell factor/lymphoid enhancer factor; DKK1: Dickkopf Wnt Signaling Pathway Inhibitor 1; PTH: Parathyroid hormone; PTH1R: Parathyroid hormone 1 receptor; SOST: Sclerostin; ROR2: receptor tyrosine kinase like orphan receptor 2; RYK: receptor-like tyrosine kinase; WNT5A: Wnt family member 5A; AKT1: AKT serine/threonine kinase 1; IP3: Inositol trisphosphate; DAAM1: Disheveled-associated activator of morphogenesis 1; JNK: c-Jun N-terminal kinases; ROCK: Rho-associated protein kinase; NFATc1: Nuclear factor of activated T-cells, cytoplasmic 1; PPAR-γ: Peroxisome proliferator-activated receptor gamma; RUNX2: Runt-related transcription factor 2. Adapted from Baron and Kneissel (2013)

Of critical importance is the osteocyte’s ability to mediate the anabolic actions of the Wnt/β-catenin signalling pathway (Tu et al. 2015). This signalling pathway is evolutionarily conserved and can be categorised into three forms: an inactive form, where β-catenin is phosphorylated and degraded by ubiquitination in the proteasome, and two active forms, termed as canonical or non-canonical (Fig. 2). It is activated upon mechanical loading e.g., generated from exercise to initiate osteogenesis and bone formation (Krishnan et al. 2006), either by direct stimulation of the bone transcription factor RUNX2 (Gaur et al. 2005) or by crosstalking with PTH or morphogenetic proteins (BMPs) signalling pathways (Baron and Kneissel 2013; Gardinier et al. 2016). A recent investigation showed that circulating PTH, generated from physical activity, led to downregulation of sclerostin (an anti-anabolic bone protein) in osteocytes (Gardinier et al. 2016) was accompanied by significant upregulation of fibroblast growth factor-23 (FGF-23) expression (Gardinier et al. 2016), a growth factor governing phosphatase homoeostasis and vitamin D metabolism (Quarles 2012). Collectively, these findings demostrate the vital role of osteocyte Wnt/β-catenin signalling in the bone adaptation to exercise.

Exercise could be a means to maintain or enhance a specific health outcome, such as maximising bone accretion and/or improving bone strength. Bone adaptation to exercise is initiated by muscle contraction and ground-reaction forces (Sharkey et al. 1995). Bone traits, such as BMD, strength and architecture, change and adapt to help the skeleton to cope with the loading environment while preventing injuries. To illustrate the bone adaptation response, athletes undertaking intermittent high impact exercise (Olympic fencers as just one example) exhibit higher densities of cortical and trabecular bone than matched controls (Chang et al. 2009). Similarly, in athlete groups where a highly active limb can be compared to a less active limb, such as the racket arm versus non-racket arm of tennis players (Haapasalo et al. 2000; Ireland et al. 2013) or in the throwing arm vs non-throwing arm of baseball players (Warden et al. 2014), there is a greater bone mass observed in the more active limb. Conversely, 6-months of spaceflight results in a 10% loss in the BMD of astronauts living under zero gravity conditions, where gravitational mechanical loading and, therefore, ground-reaction forces are missing (Sibonga 2013).

Upon beginning exercise, the skeleton is exposed to different types of strains (deformation of tissue) generated from compression, tensile and torsional forces, and shear stress. Diferent types of strains can occur at the same time and in the same bone (Judex et al. 2009). In this study, a compression strain occurred at 2500 μs on one side of the bone and a tensile strain of 2000 μs on the other side. It is also established that running generates tibial strains 2–3 times higher than walking (Burr et al. 1996) and walking higher than cycling (Milgrom et al. 2000). The optimal magnitude and frequency to initiate an osteogenic response in humans is still uncertain as most studies are undertaken in animals. On the other side, the optimal exercise to induce osteogenesis and bone anabolism is likely to change according to age, sex, the individual (Weaver et al. 2016) and even skeletal site, suggesting that only a personalised approach would provide the precision information to design the optimal osteogenic exercise regimen.

## Bone adaption to exercise across the lifespan

### Exercise interventions during childhood and adolescence

The promotion of physical exercise and healthy eating habits during bone development maximises the chances of accruing bone, potentially delaying the onset of osteoporosis in later life. Such a perspective is supported by longitudinal studies showing that individuals who were active during childhood had 8–10% greater hip bone mineral content (BMC) in adulthood (age 23–30 years) than their sedentary counterparts (Baxter-Jones et al. 2008). A more recent longitudinal trial showed that children engaged in school-based exercise interventions for 9 months had higher whole-body (6.2%), femoral neck (8.1%) and total hip (7.7%) BMC compared with their non-exercising counterparts (Meyer et al. 2013). Three years after ceasing the intervention, the benefits persisted, with a sustained 7–8% increment of BMC in the femoral neck and total hip of conditioned individuals (Meyer et al. 2013). A cross-sectional study investigating the long-term benefits of performing upper body exercise (ball throwing) suggested that half of the benefit in bone size and one-third of the benefit in bone strength was kept throughout life (Warden et al. 2014). Tveit et al. (2015) conducted a cross-sectional, cohort study investigating the long-term, 30 years after retirement, effects of soccer on BMD, bone structure and fracture risk. They showed that exercise generated higher BMD’s, larger bones and a lower fracture risk in former athletes after retirement (Tveit et al. 2015).

Peak bone mass (PBM) is regarded as a significant predictor of future osteoporosis and fracture risk (Specker et al. 2010). Bioinformatics’ and meta-analyses calculations have estimated that a 10% increase in PBM would delay the onset of osteoporosis by 13 years (Hernandez et al. 2003) and reduce fracture risk, resulting from osteoporosis, by up to 50% in post-menopausal women (Marshall et al. 1996). A 6.4% decrease in bone mass in childhood has been associated with a twofold increase fracture risk during adulthood (Boreham and McKay 2011). This evidence suggests that exercise interventions, spanning childhood and adolescence, are effective, even after the activity has ceased, although the timing of initiation may be important. An interesting recent study has also suggested that the age at which children first start walking might influence their bone strength in later life (Ireland et al. 2017). Ireland et al. (2017) examined the association betweent walking age (obtained at 2 years old) and bone outcomes determined by DXA and pQCT (between the ages of 60 and 64 years old). Later independent walking age was associated with lower height-adjusted hip, spine and distal radius BMC in men, suggesting that the ability to mechanically load the skeleton early during bone development might be important in the development of good bone health.

A systematic review addressing bone mineral changes in response to weight-bearing exercise (e.g., ball games dancing, jumping, and others) proposed that bone adaptations peak during early puberty (MacKelvie et al. 2002). More specifically, they showed that weight-bearing exercise during childhood had a positive effect on bone strength, while exercise undertaken during prepubertal and peripubertal ages caused an increment in bone mineral accrual (MacKelvie et al. 2002). These findings were reinforced by a subsequent systematic review that analysed bone mineral accrual in children and adolescents (Hind and Burrows 2007). Despite osteogenesis and bone anabolism being more pronounced during the peripubertal stage, the ideal modality or training regimen to optimise bone mass accrual remains to be elucidated.

### Exercise interventions during adulthood

Adults might also also benefit from bone-loading exercise, but systematic reviews and meta-analyses on the topic (Bolam et al. 2015; Hamilton et al. 2010; Martyn-St James and Carroll 2010) suggest that this might occur to a lesser extent than in children and adolescents (Hind and Burrows, 2007; Nogueira et al. 2014). Nonetheless, Heinonen et al. showed that pre-menopausal women, aged 35–45 years, who performed a high-impact exercise regimen, consisting of jump and step training for 18-months, had progressive increases in BMD at the femoral neck (a load bearing site) when compared with inactive controls (Heinonen et al. 1996). A meta-analysis, of randomised controlled exercise trials lasting 24 weeks, also showed improvements in femoral neck and lumbar spine BMD (Kelley et al. 2013). Bassey et al. (1998) examined bone accrual after a 12-month exercise training intervention in both pre- and post-menopausal women. Training consisted of vertical jumping, 6 times per week, and resulted in a 2.8% increase in femoral BMD in pre-menopausal women, whereas no improvements were shown in post-menopausal women after 12- or 18-months of training and hormone replacement therapy (Bassey et al. 1998). The inability of post-menopausal women to accrue bone mass after high impact training was later confirmed by a 12-month randomised controlled trial on the effect of weight-bearing jumping and oral alendronate, alone or in combination, on bone mass and structure (Uusi-Rasi et al. 2003). Exercise alone or in combination with alendronate had no effect on bone mass at the femoral neck or lumbar spine (Uusi-Rasi et al. 2003). The “anabolic resistance” to exercise shown in post-menopausal women likely results, at least in part, from depleted oestrogen levels (Ji and Yu 2015). Oestrogen is a pleiotropic hormone, with a vital role in skeletal growth and bone homoeostasis, as well as in sexual dimorphism and reproduction (Weitzmann and Pacifici 2006). All bone cells have oestrogen receptors and when circulating levels of oestrogen drop, Wnt/β-catenin and the oestrogen ERβ/GSK-3β-dependent signalling pathways are attenuated, leading to reduced osteoblastic proliferation (Yin et al. 2015). Attenuation of these signalling pathways, with concomitant diminished osteoblastic proliferation, is thought to cause the lack of responsiveness of post-menopausal women to bone-loading exercises (Yin et al. 2015).

Although these studies have shown that osteogenic and bone anabolic effects, resulting from exercise, are less pronounced or are even negligible when coupled with oestrogen depletion, post-menopausal women are strongly advised to undertake exercise. Exercise, of the right type, might well contribute to BMD preservation, presumably by maintaining cortical and trabecular volumetric BMD (Polidoulis et al. 2011), and by contributing to bone strength by means of cortical bone thickening (Uusi-Rasi et al. 2003). Among the exercise modalities tested in this population, walking provided modest benefits, due to the minor mechanical load exerted on the skeleton, while resistance and multi-component exercise programmes, encompassing strength, aerobic, and whole-body vibration exercises, were more effective in mitigating the loss of bone mass (Gomez-Cabello et al. 2012).

### Exercise interventions during older age

Studies investigating exercise on bone health in older people (50s and above) are scarce. A comparative study, which enrolled men and women in their early 50s, demonstrated that after 24 weeks of moderate strength or high intensity training, men that undertook the high intensity program gained 1.9% BMD in the spine, while women did not (Maddalozzo and Snow 2000). Allison et al. (2015) conducted a 12-month randomised controlled trial in male participants, aged 65–80 years, who performed unilateral hopping exercise, whilst the other leg remained as an inactive control. In this trial, computer tomography (CT) and DXA measurements demonstrated that unilateral hopping caused an increase in BMC in both legs, with the trained leg depicting localised changes in the proximal femur. Cortical BMC at the trochanter increased more in the exercising than in the control leg, which is thought to be important for the structural integrity of the bone (Allison et al. 2015). A similar training programme, carried out over 12-months in men aged 65–80 years, showed increased femoral neck BMD, BMC and geometry (Allison et al. 2013).

Exercise might contribute to bone health by augmenting bone mass and bone strength during younger age and by mitigating age-related bone loss. In practice, however, this statement might be an oversimplification, as there are undoubtedly several factors that mediate the effects of exercise on the bone. Current or previous habitual levels of exercise, exercise mode, type, intensity and duration will all have a significant influence on the magnitude of any effects on bone related outcomes. Recently, Ireland and Rittweger (2017) also suggested that participation motivation might also play a part in the success or failure of exercise interventions targeted at the bone (Ireland and Rittweger 2017), which is certainly an area worthy of consideration.

**Fig. 3:**
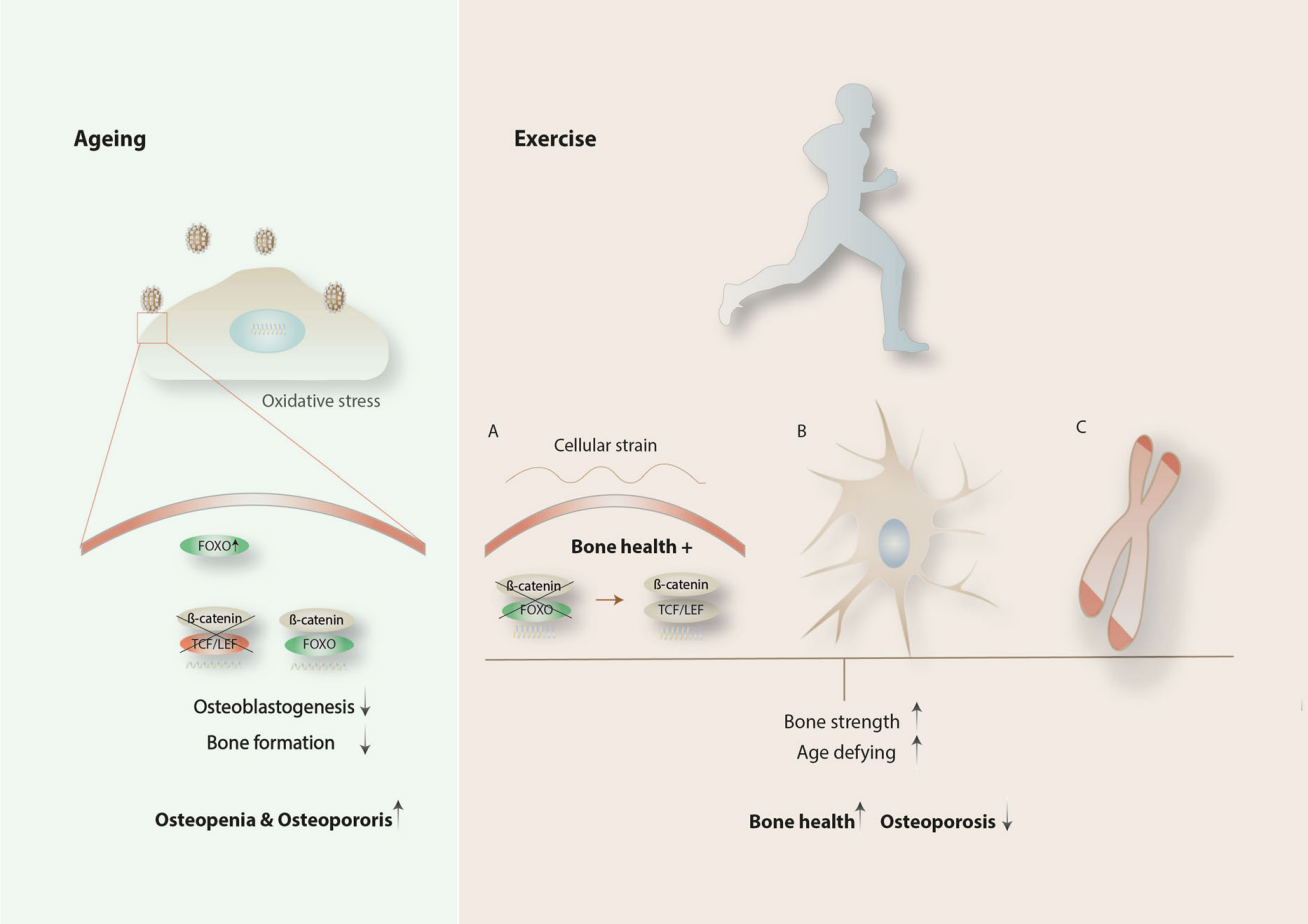
Activation of FOXO transcription signalling upon oxidative stress (left) in the context of the aged bone; Rescue of TCF/LEF transcription (**a**), prevention of osteocyte apoptosis (**b**) and prevention of telomere erosion (**c**) induced by exercise potentially contribute to bone health

Although the cellular and molecular mechanisms underpinning bone outcomes are still under investigation, the role of the Wnt/β-catenin signalling pathway both in bone health and as a target of anti-osteoporosis interventions is becoming increasing clear (Karasik et al. 2016; Korvala et al. 2012). It was reported that mechanical loading exerted on mesenchymal stem cells blocked adipogenic differentiation by rescuing β-catenin-FOXO mediated transcription to β-catenin-TCF/LEF mediated transcription (Fig. 3) (Case et al. 2013). This result was corroborated by an in vivo study involving mice, which demonstrated that exercise suppressed the accumulation of fat in the bone marrow (Styner et al. 2014), except that in this case the authors hypothesised that β-oxidation was the underpinning mechanism. Another route by which exercise might mitigate age-related bone loss is through the prevention of osteocyte apoptosis (Fonseca et al. 2011; Mann et al. 2006). This was evidenced by research conducted with ovariectomized mice exposed to exercise activity and human bone explants exposed to mechanical tension, with both providing evidence that mechanical stimulation prevented osteocyte death (Fonseca et al. 2011; Mann et al. 2006), a fact that contributes to preservation of bone strength.

Exercise, besides supporting bone health outcomes such as BMD and bone strength, provides an anti-ageing effect by virtue of preventing telomere erosion (Fig. 3) (Loprinzi et al. 2015). A longitudinal study, of 6503 participants aged 20–84 years, showed that exercise interventions prevented or delayed telomere shortening, therefore exhibiting an “age-defying” or rejuvenating action (Loprinzi et al. 2015). This study suggested that (i) a dose–response relationship exists between exercise and reduced telomere erosion and (ii) this relationship was significant in participants aged 40–64 years. According to this, undertaking exercise after the fourth decade of life appears to improve systemic health on account of the counter-ageing effect provided. This systemic effect may mitigate ageing and accordingly age-related bone loss and age-related osteoporosis. Notably, individuals with osteoporosis exhibit shorter telomeres than healthy ones (Valdes et al. 2007), a fact that supports the notion that preventing or delaying systemic ageing is beneficial to bone health. Due to the progress of molecular biology, it is possible that bone health may also now be appraised by the assessment of telomere length given that leucocyte telomere shortening correlates with lower BMD at the lumbar spine, femoral neck and total hip (Nielsen et al. 2015).

Beside the mechanisms illustrated here, we acknowledge that exercise might contribute to bone health through other routes as, for example, changes in hormone levels or by targeting signalling pathways other than Wnt/β-catenin signalling, such as the BMP or RANK/RANKL.

Exercise is also linked with epigenetic modifications, in particular, changes in DNA methylation patterns and gene expression (Brunet and Berger 2014; Jung and Pfeifer 2015; Rönn et al. 2013). DNA methylation is an epigenetic modification typically leading to long-term gene repression, achieved by the addition of a methyl group to the five position of a cytosine ring (Cedar and Bergman 2009). The relationship between exercise and DNA methylation was demonstrated in an epidemiological study comprising two groups; healthy volunteers and type II diabetics (Rönn et al. 2013). In this study, participants performed spinning and aerobic exercise over a 6-month period, with an average attendance of 1.8 times per week. DNA methylation changed in participants from both groups; more specifically in 7663 genes, one-third of which showed altered mRNA expression levels (Rönn et al. 2013). In another study, young sedentary participants of both sexes were exposed to acute bouts of exercise to ascertain whether acute exercise could change DNA methylation patterns. DNA was hypomethylated in skeletal muscle, in a dose-responsive fashion, with similar findings in mouse muscles 45 min after ex vivo contraction, both suggesting a putative role of exercise in epigenetic modification through DNA methylation (Barrès et al. 2012). The causal relationship between exercise and changes in DNA methylation was further corroborated by an investigation enrolling young male and female individuals in a 3-month fully supervised one-legged exercise training programme. Here, DNA methylation patterns changed in 4919 sites across the genome of the trained leg group (Lindholm et al. 2014). These epigenetic studies allowed identification of changes in DNA methylation patterns resulting from exercise on healthy, type II diabetic and young sedentary populations. To undertake similar studies in the older individual might reveal an age reversing epigenetic signature induced by exercise that might be utilised as a technique to asses not only bone health but also the effect of exercise in older individuals with chronic bone diseases.

## Conclusions

Osteoporosis is a bone metabolic disease that prevails in post-menopausal women. The first line of treatment relies on anti-osteoporotic drugs, particularly bisphosphonates, although this type of therapy can only be provided for a limited period of time and the benefits are transient. Exercise has the potential to provide a means of non-pharmacological intervention, with long-lasting effects that can delay the onset of osteoporosis, particularly if performed during the peripubertal stage, a time during which exercise-induced osteogenesis and bone anabolism is more accentuated. There are no current data, however, to directly compare appropriate exercise with pharmacological interventions designed to prevent bone loss or increase bone mass. These studies are urgently required to determine the extent to which exercise may or may not be able to provide a sole (highly unlikely) or adjunct therapeutic intervention against osteoporosis.

Exercise might be recommended following the menopause to mitigate the age- and menopausal-related loss of bone and to strengthen cortical bone. During growth and development PBM should be maximised, with exercise potentially providing a means to help achieve this. During middle- and older-age, weight-bearing exercises should be performed to maintain bone mass and increase bone strength. It remains largely unknown, however, what the best type of exercise is in terms of mode, type, intensity and duration to maximise bone responses. It is likely that any exercise would need to be high-intensity, high-impact, multidirectional and possibly unaccustomed in order to optimise osteogenic responses, but this approach might not be suitable for all.

**Table:** Glossary

	Acronym	Definition
Dual-energy X-ray absorptiometry	DXA	Standard methods to measure BMD. Two X-ray beams with different energy levels are conveyed to the patient’s bone. After subtracting the signal from soft tissue, the obtained absorption values allow to estimate bone BMD
Computed tomography	CT	Imagining technique that allows obtaining detailed scans of areas inside the body
Bone mineral density	BMD	Refers to the amount of mineral matter per square centimetre of bone. BMD is utilised as predictor of osteoporosis and fracture risk. Parameter utilised to estimate bone strength
Aerial bone mineral density	aBMD	It is a reasonable estimate of BMC and bone strength, not an accurate measurement of true bone mineral density, which is mass divided by volume. Parameter utilised to estimate bone strength
Bone mineral content	BMC	Estimated by DXA, these measurements reflect BMD at specific body parts, spine, hip, wrist, femur or other selected part of the skeleton. The values obtained are divided by the surface area of the bone being measure to create BMD
Peak bone mass	PBM	Amount of bone gained by the time a stable skeletal state has been attained. At a population level, peak bone mass reflects the maximum bone mass attained across the lifespan. It is a predictor of osteoporosis
Volumetric peak bone mass	vPBM	Refers the amount of peak bone mineral content per cubic centimetre of bone
